# Neural Mechanisms of Role Reversal in Improvisational Music Psychodrama: An fNIRS Hyperscanning Study

**DOI:** 10.3390/brainsci15111235

**Published:** 2025-11-18

**Authors:** Ying Wang, Kangzhou Peng, Yueqing Zhang, Yuan Yao, Zhen Zhang, Fupei Zhao, Maoping Zheng

**Affiliations:** 1 Department of Psychology, Southwest University, No. 2 Tiansheng Road, Chongqing 400715, China; sanjingzhai@163.com (Y.W.); zzhen2025@126.com (Z.Z.); 18275603911@163.com (F.Z.); 2 School of Foreign Languages, Chongqing Normal University, No. 37, University Town Middle Road, Chongqing 401331, China; 20131909@cqnu.edu.cn; 3 The Communist Youth League Committee, Chongqing Normal University, No. 37, University Town Middle Road, Chongqing 401331, China; 13193137773@163.com; 4 Department of Psychology, Suzhou University of Science and Technology, No. 99 Xuefu Road, Suzhou 215009, China; lisayuan@zju.edu.cn

**Keywords:** improvisational music psychodrama, role reversal, functional near-infrared spectroscopy (fNIRS), inter-brain synchrony, hyperscanning

## Abstract

**Background:** The neural mechanisms underlying role-playing and role reversal in improvisational music psychodrama remain poorly understood. This study aimed to investigate the specific neural correlates and behavioral associations of these processes. **Methods:** Using functional near-infrared spectroscopy (fNIRS) hyperscanning, inter-brain synchrony (IBS) was examined in 46 dyads of participants during improvisational role-playing and role reversal tasks. Behavioral changes were assessed using a negative emotion questionnaire. **Results:** Behavioral results indicated a significant reduction in negative emotion scores following the intervention compared to baseline. At the neural level, the role reversal task elicited significantly stronger activation in the right frontopolar area and induced higher IBS in the right supramarginal gyrus area compared to the role-playing task. **Conclusions:** The findings demonstrate that role reversal is associated with distinct neural activation patterns and enhanced inter-brain coordination. Coupled with the observed reduction in negative emotions, this provides empirical evidence elucidating the mechanisms underlying music psychodrama.

## 1. Introduction

Psychodrama, developed by psychiatrist Jacob Levy Moreno in the 1930s, is defined as “a scientific approach to exploring truth through dramatic methods.” It is structured around five core elements: the director, the protagonist, auxiliary egos, the audience, and the stage, with role-playing and role reversal being among its central techniques [1,2,3]. A key process in psychodrama, particularly role reversal, promotes cognitive-affective restructuring by requiring individuals to shift between their own perspective and that of another. This mechanism demands not only the external imitation of others but also the internal adoption of their viewpoints, thereby challenging and potentially modifying one’s pre-existing cognitive schemas and emotional responses [4]. Since the 1950s, international research on psychodrama has primarily focused on two areas: empirical evaluation and theoretical development. Quantitative studies, often using randomized controlled trials, have assessed its efficacy in treating conditions such as eating disorders, substance addiction, and depression [5,6,7]. Meanwhile, theoretical reviews have sought to clarify its therapeutic mechanisms and refine its conceptual framework [8].

Both music therapy and psychodrama foster spontaneity and emotional expression. In 1980, Joseph J. Moreno formally established this integrated discipline through his article “Music Psychodrama: A New Direction in Music Therapy.” His foundational work, Acting in Your Inner Music, combined improvisational music playing and imagery with traditional psychodrama techniques to achieve synergistic therapeutic effects [9,10]. When psychodrama is integrated with musical improvisation, this cognitive-affective process is deepened and expanded. Music provides a pre-linguistic, embodied channel of expression, enabling participants to externalize and regulate complex emotions emerging during role reversal through rhythm and melody in real time [11]. Musical improvisation is a form of musical creativity conceptualized as participatory sense-making, where musical choices emerge through continuous feedback within a dynamically changing context [12]. Biasutti and Frezza identified five key dimensions—anticipation, emotional communication, flow, feedback, and repertoire use—and emphasized the importance of musical practice and foundational skills for fluent, expressive improvisation [13]. Empirical findings show that musical improvisation can be either structured (for trained musicians) or free (for untrained individuals). Studies have revealed significant interactions between instrument type and skill level, as well as enhanced creativity during mind-wandering compared to focused states [13,14]. In therapeutic and educational settings, musical improvisation not only facilitates emotional expression and activates neural reward systems in children, but also supports their self-expression and social reintegration, and enhances memory performance in children more effectively than musical reproduction activities [15,16].

Standard psychodrama relies primarily on verbal interaction, utilizing techniques such as role-playing and role reversal to facilitate emotional regulation and cognitive restructuring. In contrast, music psychodrama incorporates improvisational music performance—either preceding, following, or concurrently with verbal interaction—using rhythm to deepen emotional expression and enhance interpersonal resonance [9]. Thus, integrating the core psychodrama technique of role reversal with improvisational music performance provides a multisensory, experiential field that transcends verbal communication. This approach deepens and expands the therapeutic process of traditional psychodrama by revealing clients’ inner experiences—including unspoken thoughts, emotions, and imagined perspectives [1].

The advent of hyperscanning has transformed social cognitive neuroscience by enabling the simultaneous measurement of brain activity across individuals, thus revealing mechanisms of inter-brain synchrony (IBS) during social interaction [17,18]. Functional near-infrared spectroscopy (fNIRS), though lower in temporal and spatial resolution than electroencephalography (EEG) and functional magnetic resonance imaging (fMRI), offers advantages such as high ecological validity, low cost, and strong motion tolerance, making it ideal for studying naturalistic cooperative behaviors [19,20]. Hyperscanning dynamically captures IBS—the coordination of neural activity between interacting individuals—particularly in joint musical performance and appreciation. IBS is typically quantified using correlation, coherence, phase synchronization, and causality metrics, reflecting the strength and timing of neural coupling during shared tasks [21,22,23].

This study constructs its analytical framework based on three interrelated theoretical pillars: predictive processing theory elucidates the mechanism through which role reversal induces cognitive reappraisal; role theory reveals its function in updating self-other models; and inter-brain synchronization theory clarifies how musical improvisation enhances this process via neural coupling. Together, these theories form a multi-level explanatory framework for understanding the efficacy of role reversal in musical psychodrama. Under Clark’s predictive processing framework, the brain continuously generates top-down predictions through internal models and compares them with incoming sensory inputs. The mismatch between predictions and actual evidence yields prediction errors [24]. In depression, rigid negative priors and cognitive immunization maintain pessimistic expectations, thereby reinforcing a maladaptive cycle [25]. Within this framework, role reversal acts as a potent mechanism for belief updating. By physically adopting another’s perspective, the individual is exposed to sensory evidence that contradicts their negative prior beliefs. This conscious shift in perspective generates significant prediction errors, which, within a supportive therapeutic environment, can drive cognitive reappraisal and promote the recalibration of internal models with external reality.

Role reversal, often regarded as the engine of psychodrama, is grounded in classical role theory and supported by empirical evidence demonstrating its robust therapeutic efficacy. Meta-analyses, systematic reviews, and studies on the neurobiological mechanisms of psychodrama have consistently demonstrated that (this technique, e.g., role reversal) ranks among the most efficacious and frequently utilized core techniques. Its application is associated with significant improvements in mental health and interpersonal functioning, as well as the facilitation of self-other model updating [3,26,27,28]. In this process, two individuals enact each other’s roles within an interpersonal context, temporarily embodying the other’s perspective before returning to their primary selves [29]. This practice extends beyond mere behavioral imitation, constituting a profound cognitive-affective exercise that restructures representations of both self and other. Compared to a first-person condition, although role reversal does not enhance insight into one’s own behavior, it heightens awareness of others’ actions and strengthens the sense of connection with them. Through psychodramatic enactment, individuals gain the opportunity to “interpret themselves to themselves,” thereby transcending the conventional subject-object dichotomy [30]. Through dialogical interaction between the protagonist and auxiliary egos, role reversal promotes the integration of discrepant social feedback into a coherent and adaptive self-model.

Music psychodrama provides a unique paradigm for investigating the neural mechanisms of social coordination by integrating musical improvisation with role reversal. Within this integrated framework, the inherent non-verbal feedback loops of musical improvisation directly foster behavioral and emotional synchrony, while role reversal drives cognitive-affective restructuring through perspective-taking. At the neural level, this process is reflected in enhanced inter-brain synchrony, particularly within the frontal-temporal-parietal network associated with social cognition [31]. We thus propose that the synergy between musical improvisation and role reversal leverages the brain’s innate capacity for interpersonal neural coupling. By strengthening inter-brain synchrony, this integrated approach facilitates perspective-taking and emotion regulation, thereby creating an optimized neural environment for cognitive reappraisal and updating of self-other models.

This neural mechanism aligns with the psychodynamic basis of music psychodrama. Rooted in Moreno’s theory, by aligning with the protagonist’s emotional state, improvised music not only establishes a pathway that bypasses intellectualized defenses but also directly engages the client at affective and right-hemispheric levels [32,33]. Neuroscientific evidence further indicates that such musical engagement modulates emotional networks involving the prefrontal and temporal cortices, together with the limbic system [34,35]. Thus, music psychodrama serves as a translational framework that bridges psychodynamic constructs (e.g., unconscious emotional expression) with measurable neural activity: by eliciting inter-brain synchrony through musical collaboration, it fosters cognitive-affective integration at the neural level.

Although psychodrama intervention has been demonstrated to be associated with reduced negative emotions, the underlying mechanisms of music psychodrama remain underexplored. Building on findings from role reversal and musical improvisation research, the former disrupts maladaptive cognitive cycles through perspective-shifting, while the latter facilitates emotional expression, reduces anxiety, and enhances social engagement without reliance on verbal processing [36,37]. Yet, empirical evidence on the neural basis of musical psychodrama remains scarce. This study proposes that integrating role reversal and musical improvisation within musical psychodrama fosters emotional release and self-regulation through experiential interaction. We introduce an innovative paradigm—improvisational role reversal with the double technique—to investigate its neural correlates. Musical improvisation enhances intuitive affective expression and engagement, while the double technique externalizes and integrates implicit emotions [38,39].

Neuroimaging evidence indicates that role reversal engages prefrontal and mirror neuron systems, supporting empathy, perspective-taking, and self–other integration [40,41,42,43]. Furthermore, musical improvisation engages the prefrontal (PFC) and tem-poroparietal regions (TPJ), reflecting coordinated activity in large-scale brain networks underlying cognitive control and spontaneous thought. This process enables real-time adaptation to the ongoing environmental context through continuous monitoring and feedback of sensory states associated with internal plans and goals [44,45]. Building on these findings, and informed by recent hyperscanning studies underscoring the importance of the right hemisphere—particularly PFC and TPJ—in achieving IBS during interpersonal interaction and emotion regulation, this study focuses its analysis on right-hemispheric mechanisms [46,47]. Accordingly, we propose the following hypotheses:

**H1.**  *In the context of musical improvisation, the role reversal condition will elicit stronger intra-brain activation in the right frontopolar region (FP.R) compared to the role-playing condition.*

**H2.**  *The role reversal condition will induce higher inter-brain synchrony in the right supramarginal gyrus (SMG.R) relative to the role-playing condition.*

## 2. Methods

### 2.1. Participants

A priori power analysis was performed using G*Power software (version 3.1.9.7). With a statistical power of 0.95, significance level of α = 0.05, and aiming to detect at least a medium effect size (Cohen’s f = 0.25) in a within-subjects design, the analysis indicated a required sample size of 84 participants (42 dyads). To account for potential channel signal loss during fNIRS data preprocessing, we ultimately recruited 46 dyads (The total sample size was N = 92; comprising 36 female-female dyads and 10 female-male dyads) with social anxiety traits through convenience sampling. Participants aged between 18 and 28 years (The mean age was M = 20.13 years).

According to the Manual of Mental Health Rating Scales (Revised Edition), this study employed the Interaction Anxiousness Scale (IAS) to assess social anxiety levels. All participants scored ≥39 (total score range: 15–75) on this widely recognized clinical instrument for social anxiety evaluation.

Developed by Leary in 1983, the IAS comprises 15 items rated on a 5-point Likert scale from 1 (“not at all characteristic of me”) to 5 (“extremely characteristic of me”). The scale demonstrates excellent psychometric properties, including test-retest reliability of 0.80 over 8 weeks, item-total correlations all above 0.45, and Cronbach’s α exceeding 0.87, confirming high internal consistency and temporal stability [48,49].

This study focused on individuals with social anxiety due to their more pronounced emotional and physiological responses during social interactions, which may lead to more detectable changes in brain activity and behavior during psychodrama interventions such as role reversal. Furthermore, by restricting the sample to this homogeneous clinical population, we could better control for baseline differences and more clearly attribute changes in inter-brain synchronization to the experimental manipulation itself. Role reversal specifically targets core deficits in social anxiety—rigid self-perception and empathy deficiencies. One of the long-term objectives of this study, which integrates psychodrama and neuroscience, is to provide neurobiological evidence and novel insights for the psychotherapeutic treatment of social anxiety disorder. Although this approach limits immediate generalizability, it establishes a solid theoretical foundation and crucial preliminary evidence for subsequent validation studies in more heterogeneous populations.

All participants self-reported as right-handed, with no history of neurological or psychiatric disorders, and had normal or corrected-to-normal vision. Prior to the experiment, all participants received 30 min of training in basic psychodrama techniques and musical improvisation to ensure their ability to adequately complete the experimental tasks. The study was approved by the Ethics Committee of Southwest University (Approval No: H25091). All participants provided written informed consent and received ¥50 RMB as compensation for their participation.

### 2.2. Experimental Tasks and Procedure

The experiment was conducted in a sound-attenuated laboratory (ambient noise < 40 dB, standard illumination, comfortable temperature) with continuous video recording for behavioral analysis. Drums and tambourines were selected as the improvisational instruments due to their ease of use and capacity for direct emotional expression through rhythm. No constraints were imposed on rhythm or tempo during performances to maintain natural interaction. All participants completed both experimental conditions—role-playing and role reversal—in a fixed (non-randomized) sequence, as required by the music psychodrama protocol where role-playing must precede role reversal.

The procedure involved four trained personnel: one protagonist, one auxiliary ego, and two professionally trained doubles. Role-playing served as the foundational phase for scenario construction and baseline establishment, wherein participants “became” themselves or a designated role. During this phase, the protagonist and auxiliary ego faced each other while engaging in musical improvisation. Role reversal, the core perspective-shifting behavior emphasizing “stepping into another’s shoes,” involved the protagonist and auxiliary ego switching roles while continuing improvisational music-making. To accommodate potential verbal communication difficulties in socially anxious participants, the doubles primarily provided verbal supplementation and only engaged in instrumental improvisation when emotional elicitation was needed.

**Figure 1:**
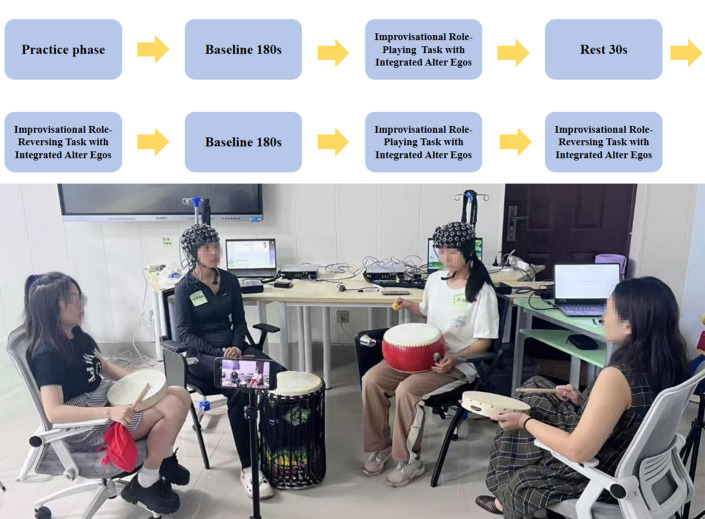
Schematic diagram of the experimental design. Practice Phase → Resting Phase → Formal Experimental Phase → 30-s Rest Period (followed by one additional cycle). During the practice phase, the two participants first completed a 30-min joint practice session to familiarize themselves with the theme of improvisational role-playing. This was followed by a 3-min resting period, after which the formal experiment commenced. In the formal task, the protagonist first performed musical improvisation, followed by verbal supplementation from the double. Then the auxiliary ego performed musical improvisation, again followed by verbal supplementation from the double. Each task block lasted 3 min, followed by a 30-s rest period, after which the task cycle was repeated once (The content within the blocks refers to the specific tasks).

The standardized experimental procedure (detailed in Figure 1) consisted of the following phases:

(1) Pre-experiment Preparation: After determining role-playing themes and roles, participants completed open-ended prompts on paper templates to familiarize themselves with assigned themes and roles, followed by training in music psychodrama intervention.
(2) Baseline (3 min): Participants sat quietly with eyes closed, performing neutral breathing exercises.
(3) Task Phase (6 min, comprising two 3-min blocks with a 30-s rest):

Role-playing: Participant A (protagonist) began with instrumental improvisation, followed by a response from Participant B (auxiliary ego). The double provided verbal support from a 45-degree position behind the protagonist/auxiliary ego, while the respective role continued instrumental playing to maintain behavioral continuity.

Role Reversal: Participants exchanged roles and functions—the original auxiliary ego became the protagonist (initiating improvisation) and the original protagonist assumed the auxiliary role (responding musically). Name tags were swapped, positions remained unchanged. The double provided verbal supplementation while both roles sustained simultaneous instrumental improvisation to avoid interaction disruption.

(4) Resting Phase (3 min): Participants sat quietly with eyes open, refraining from active task-related thinking.
(5) Pre- and post-experiment, participants rated negative emotions on a 5-point Likert scale (1 = completely inconsistent, 5 = completely consistent). The total duration of the experimental protocol (including baseline, task, and rest phases) was approximately 18.5 min, with 12 min of valid interactive recording.

The specific experimental procedure is illustrated in Figure 1.

### 2.3. fNIRS Data Acquisition

**Figure 2:**
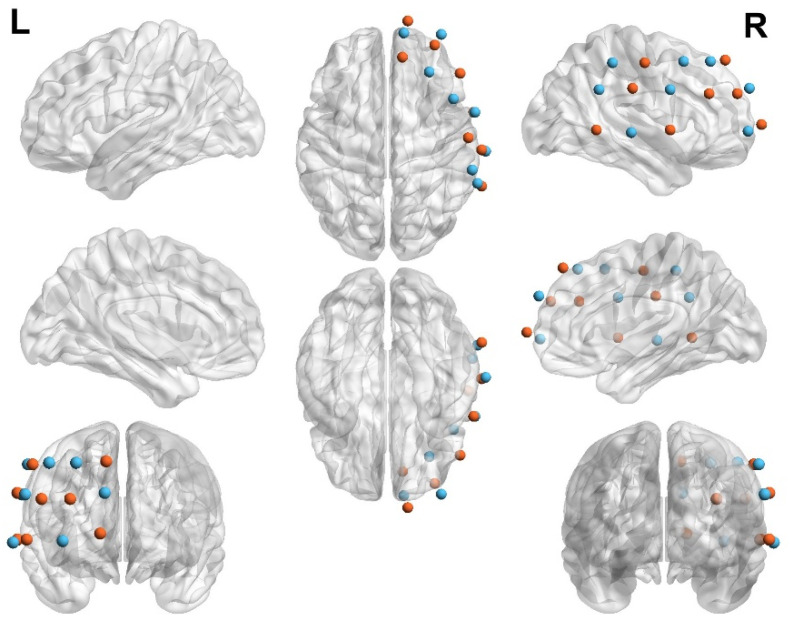
Brain region localization map. The map illustrates the regions of interest (ROIs), which are primarily concentrated in the right prefrontal cortex (PFC) and the right temporoparietal junction (TPJ).

The experimental data were acquired using a functional near-infrared spectros-copy (fNIRS) system (SHIMADZU Corporation, Kyoto, Japan; model: Lightnirs) controlled by kNIRS software (version 0.5.400). This device employs continuous-wave measurements at three wavelengths (780 nm, 805 nm, and 830 nm) to assess cortical hemodynamic activity. Changes in oxygenated hemoglobin, deoxygenated hemoglobin, and total hemoglobin concentrations were monitored using a modified Beer-Lambert law [19]. The system sampling frequency was 13.33 Hz. Each unit was equipped with 8 emitters and 8 detectors, forming 20 measurement channels on the scalp surface. The source-detector distance was set to 3 cm, with each source-detector pair defined as a measurement site for a specific brain region. These channels covered the right PFC, right dorsolateral prefrontal cortex (DLPFC), and the right TPJ. Regions of interest (ROIs) were identified using the fOLD toolbox (fNIRS Optodes’ Location Decider) in MATLAB [50]. Subsequently, the anatomical positions of the optodes and channel distribution were evaluated in a standardized 3D head model using the NIRS_SPM toolbox (http://www.nitrc.org/projects/nirs_spm/, accessed on 1 July 2025) based on MATLAB, yielding Montreal Neurological Institute (MNI) coordinates for the optodes and cortical localization probabilities for all 20 channels [51,52] (see Table A1 and Figure 2, Please refer to the Appendix A for further details.).

The decision to focus on the right hemisphere was based on three primary considerations. First, substantial neuroscientific evidence identifies the right hemisphere as dominant for social and emotional processing, playing a leading role in managing social interactions, processing nonverbal cues, emotional experience and regulation, and empathy [46,47]. Second, given the finite number of channels available with fNIRS systems, concentrating limited optode resources on theoretically relevant regions enhances experimental efficiency. Finally, restricting analysis to the right hemisphere significantly reduces the number of multiple comparisons required during statistical testing. This strategy enhances statistical sensitivity by mitigating the risk of Type II errors (i.e., failing to detect true effects) that arises from stringent multiple comparison corrections, thereby increasing the likelihood of detecting significant inter-brain synchrony or activation changes in our target brain areas.

To monitor and minimize motion artifacts, probes were securely fixed using an elastic head cap and sponge pads, and participants were instructed to limit head movement. Raw signals were monitored in real-time by the experimenter during data acquisition. During preprocessing, an automated motion artifact detection algorithm (e.g., based on the first derivative of the signal and amplitude thresholding) identified contaminated data segments, which were subsequently corrected using the Correlation-Based Signal Improvement method. Channels with persistently poor signal quality or excessive artifacts were excluded from the final analysis.

### 2.4. Data Analysis

#### 2.4.1. Behavioral Data Analysis

Participants completed both the Negative Emotion Questionnaire and the Visual Analogue Scale (commonly used in short-term intervention studies to assess pre-post changes in anxiety) before and after the music psychodrama intervention. Independent-samples *t*-tests were conducted using SPSS 26.0 to compare pre- and post-test scores, in order to determine whether the intervention effectively alleviated negative emotions.

#### 2.4.2. fNIRS Data Analysis

Preprocessing: All raw fNIRS recordings were preprocessed using the NIRS-KIT toolbox implemented in MATLAB 2024a [53]. Initially, signal quality was checked by visual inspection. Channels lacking a distinct cardiac rhythm (~1 Hz) in the wavelet spectrum were classified as invalid [54]. Overall, 96.576% of the recorded channels met the criteria and were retained for further analysis. Dyads were excluded only if more than 50% of their channels were flagged as invalid [55]; in the present dataset, no dyads were removed at this stage. Next, the optical density signals from the 20 measurement channels were converted into light intensity values according to the modified Beer–Lambert law using NIRS-KIT, and subsequently transformed into concentration changes of oxy- and deoxy-hemoglobin [53]. To address motion-related artifacts, polynomial regression was first employed to capture and subtract linear or nonlinear signal drifts. Correlation-based signal improvement (CBSI) was then applied to further correct motion-induced noise [56]. Correlation-based signal improvement (CBSI) was then applied to further correct motion-induced noise [56]. The algorithm automatically calculated the scaling factor (α = σ_HbO/σ_HbR) for each channel to restore the intrinsic negative correlation between HbO and HbR signals. Motion correction was performed separately for each dyad to ensure individualized preprocessing. Finally, to minimize systemic and environmental interference, a noise regression step was carried out to suppress global physiological signals such as scalp blood flow. A temporal band-pass filter (0.01–0.20 Hz) was subsequently applied to retain task-related fluctuations while removing slow drifts and high-frequency noise [57].

Intra-brain activation: We concentrated on oxygenated hemoglobin (HbO) responses, as HbO is considered a more reliable indicator of neural activity than deoxygenated hemoglobin (HbR) [58]. General Linear Model (GLM) and batch *t*-tests were conducted using MATLAB 2024a. To estimate task-related changes, a general linear model (GLM) was applied to each participant’s HbO time series. The GLM modeled all task blocks of the same condition jointly (i.e., pooled across repetitions) to obtain a single β estimate per condition, ensuring a stable estimation of task-related activation. In this procedure, task epochs were convolved with a canonical hemodynamic response function (HRF) to build the design matrix, and β coefficients were obtained for every experimental condition [59]. Finally, independent-sample *t*-tests were performed on the β-values derived from the role-playing and role-reversal tasks to evaluate condition-related differences in intra-brain activation, and all comparisons were corrected for multiple comparisons using the false discovery rate (FDR) method.

**Figure 3:**
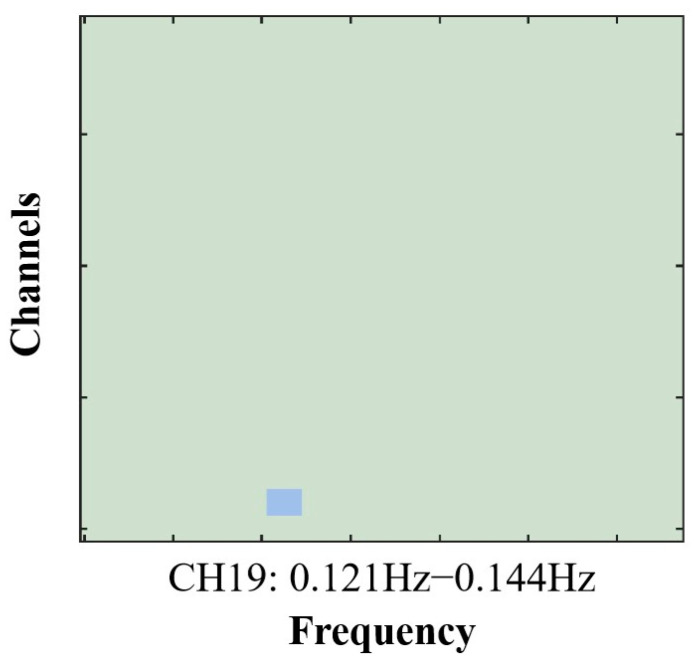
Frequency distribution map of FDR-corrected *p*-values (<0.01) for the task comparison. Inter-brain synchrony (IBS) was markedly greater in the role-reversal condition than in the role-playing condition, with the significant frequency window spanning 0.121–0.144 Hz. The blue boxes highlight the designated frequency of interest (FOI).

Inter-brain synchrony: The preprocessed HbO signals (prior to filtering) were imported into MATLAB 2024a, and inter-brain coherence was computed using the Wavelet Transform Coherence (WTC) toolbox [60]. For each dyad, coherence values were obtained across 20 channel pairs, providing a measure of the temporal correspondence between participants’ HbO time series. The resulting coherence estimates were then converted using Fisher’s Z transformation to normalize the distribution. To identify relevant frequency ranges, a data-driven approach was applied to compare coherence values across the two task conditions (role-playing vs. role-reversal) [61]. Independent-sample *t*-tests revealed a significant difference within the 0.121–0.144 Hz band, which was defined as the frequency of interest (FOI; see Figure 3). This FOI was further confirmed through post hoc FDR correction. Importantly, this range excluded major sources of physiological noise, including blood pressure oscillations (~0.1 Hz), respiratory rhythms (~0.2–0.3 Hz), and cardiac cycles (~1 Hz), thereby minimizing contamination from systemic artifacts [62]. To provide a more intuitive visualization of the statistical results, we plotted a significance frequency map (*p* < 0.01 mask), which showed that only one channel reached significance at this stringent threshold. However, when the significance threshold was relaxed to *p* < 0.05, a substantially larger number of channels exhibited significant effects, further indicating that this frequency band generally showed a robust task-related differentiation.

## 3. Results

### 3.1. Behavioral Results

The mean and standard deviation of negative emotion scores before the intervention were 21.220 ± 4.130, and after the intervention were 18.200 ± 4.350. And the mean and standard deviation of anxiety scores before the intervention were 24.74 ± 15.22, and after the intervention were 17.36 ± 11.12. An independent-samples *t*-test was performed on both the Negative Emotion Questionnaire and the Visual Analogue Scale scores, revealing a significant main effect of time (negative emotion: *t* = 3.417, *p* = 0.001, *Cohen’s d* = 0.710; anxiety: *t* = 2.655, *p* = 0.009, *Cohen’s d* = 0.550). Follow-up analyses showed that both negative emotion and anxiety scores were significantly higher at pre-test compared with post-test (negative emotion: *p* = 0.001; anxiety: *p* = 0.009).

### 3.2. Intra-Brain Activation Results

**Figure 4:**
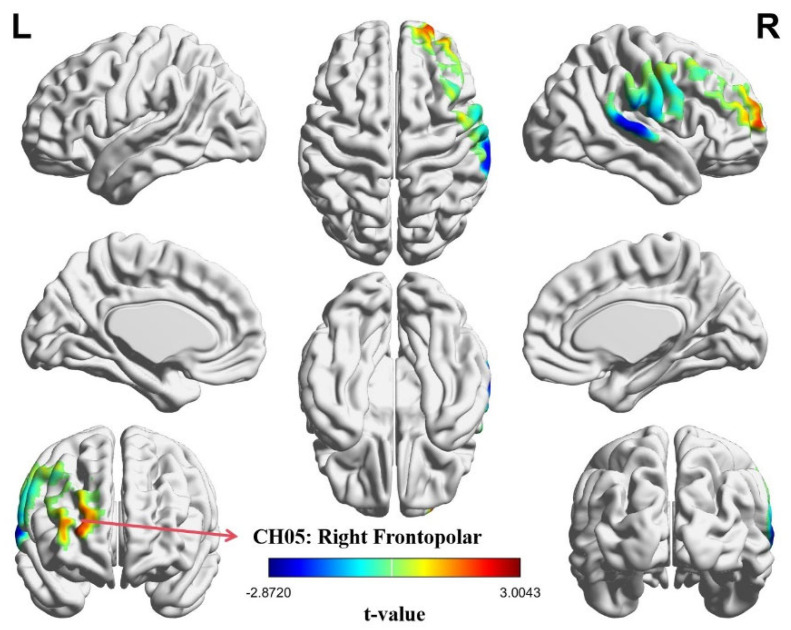
Heatmap of intra-brain activation under different task conditions. Neural activation was greater in the role-reversal task compared with the role-playing task. The highlighted red region marks the significant channel, CH05. The values represent the *t*-values of each channel. The FDR correction *p*-value threshold was set at *p* < 0.05.

Independent-sample *t*-tests were applied to assess the influence of task type on intra-brain activation across 20 measurement channels. A significant effect of task condition emerged in CH05, located in the right frontopolar region (FT.R). Specifically, CH05 showed a reliable difference (*t* = 3.004, *p* = 0.049, *Cohen’s d* = 0.630), with post hoc comparisons confirming that the role-reversal task elicited greater intra-brain activation than the role-playing task (*p* = 0.049). A visualization of intra-brain activation patterns is displayed in the heatmaps shown in Figure 4.

### 3.3. Inter-Brain Synchrony Results

**Figure 5:**
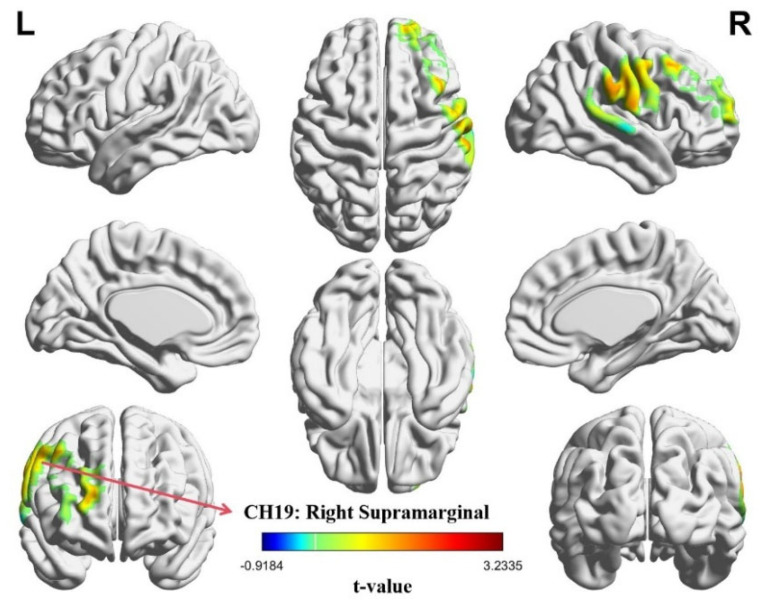
Heatmap of inter-brain synchrony (IBS) across task conditions. The role-reversal task elicited stronger IBS than the role-playing task. The red-highlighted area marks the significant channel, CH19. The values represent the *t*-values of each channel. The FDR correction *p*-value threshold was set at *p* < 0.05.

Independent-sample *t*-tests were performed to evaluate task-related differences in IBS across 20 channels. A significant effect of task type emerged in CH19, corresponding to the right supramarginal gyrus (SMG.R). Specifically, CH19 showed a reliable difference (*t* = 3.234, *p* = 0.046, Cohen’s d = 0.670), with follow-up analyses indicating that the role-reversal condition elicited stronger IBS values compared with the role-playing condition (*p* = 0.046). The spatial distribution of these effects is illustrated in the IBS heatmaps shown in Figure 5.

## 4. Discussion

Although prior studies have examined cerebral activation and inter-brain synchronization during psychodramatic role-playing and role-reversal, the distinct neural mechanisms underlying improvisational music psychodrama remain unclear. To address this gap, the present study employed an improvisational role-reversal task incorporating the alter ego technique and fNIRS hyperscanning to explore how musical psychodrama regulates negative emotions and their neural correlates. Results revealed a significant reduction in negative affect following the intervention. At the individual level, the role-reversal task elicited stronger intra-brain activation, particularly in the FT.R; at the inter-brain level, it induced higher synchronization in the SMG.R. Activation of the PFT suggests enhanced cognitive control during emotion regulation, which is associated with improvements in hyperarousal symptoms and overall psychological well-being. Furthermore, FT.R, in particular, has been linked to the neural benefits of brief relaxation [63,64]. However, it should be noted that this increased activation may not solely stem from empathic processes but could also reflect elevated cognitive load due to greater task complexity [65]. Role reversal requires individuals to maintain self-representation while rapidly constructing and sustaining a simulation of another’s mental state, potentially significantly increasing working memory demands. Additionally, this activation may relate to intensified self-monitoring—a metacognitive process that guides self-awareness and provides critical input for self-regulatory control mechanisms [66]. Similarly, enhanced IBS in the SMG.R was associated with neural activity in mirror-related networks [67,68], suggesting that dyads achieved a higher level of neural coordination in action understanding and intention inference. This finding supports the hypothesis that role reversal enhances interpersonal understanding by promoting action-perception coupling between interaction partners. Nevertheless, it is important to consider that such synchrony enhancement could also be influenced by structural task factors, including increased interaction duration or enhanced nonverbal cues, which should be further controlled in future studies.

A clear distinction should be made between the respective roles of musical improvisation and role reversal: while role reversal primarily facilitates the updating of cognitive models through perspective-taking, musical improvisation enhances emotional expression and interactive synchrony via non-verbal channels. The combination of the two may produce a synergistic effect: music creates a safe environment for emotional expression, while role reversal promotes cognitive restructuring within this context. Research indicates that musical improvisation yields therapeutic benefits across various clinical populations, including the reduction of neurological and psychological symptoms, decreased stress and anxiety, and improved communication and joint attention in children with autism. Free improvisation has been shown to significantly reduce performance anxiety [69], and improvisational psychodynamic music therapy alleviates depression and anxiety symptoms by modulating cortical activity in frontotemporal and temporoparietal regions [70]. Role reversal effectively helps children understand and express emotions, enhancing their emotional comprehension, empathy, and social interaction abilities [71]. In psychodrama, by integrating role theory with object relations theory, this approach facilitates the development of an integrated self, effectively alleviates intrapsychic conflicts and anxiety/guilt feelings, and thereby enhances spontaneity and creativity among group members [72]. Additionally, the double technique activates the mirror neuron system through observation and imitation, promoting intuitive understanding, emotional resonance, and perceived social support, thereby reducing feelings of loneliness and negative emotions [73]. Collectively, these findings provide empirical support for Hypothesis 1: music psychodrama based on role reversal is associated with a significant reduction in participants’ negative emotions, confirming its efficacy in emotion regulation and psychological support.

This study yielded two principal findings. First, compared to the improvisational role-playing task, the improvisational role-reversal task elicited significantly stronger intra-brain activation in the FT.R. The incorporation of musical improvisation introduced an element of emotional memory updating, which aligns with the established role of the PFC in improvisation and face-to-face interaction [74,75]. This finding is also consistent with prior evidence that musical improvisation enhances connectivity within emotion and reward networks while modulating activity in the PFC and TPJ [44,45]. Second, the improvisational role-reversal condition induced significantly higher inter-brain synchrony in the SMG.R This effect reflects the engagement of the mirror neuron system, which supports imitation, intention inference, and empathy. The SMG.R acts as a higher-order hub integrating sensorimotor information for social cognition, corroborating existing evidence that both the SMG and inferior frontal gyri exhibit sensorimotor coupling during self- and other-related actions [68]. Collectively, this study provides a more specific integration with the predictive processing framework, informing mechanisms of model updating and prediction error resolution. The results suggest that improvisational role reversal may facilitate internal model updating by introducing sensory evidence (prediction errors) that conflicts with an individual’s pre-existing models. Activation in the FT.R likely supports the comparison and integration of old and new models, while enhanced synchrony in the SMG.R. reflects neural coordination between interacting partners during mutual prediction error minimization. These findings together offer empirical support for Hypothesis 2.

This study has several limitations that warrant consideration in the context of future research directions. First, the relatively small sample size and homogeneous gender distribution highlight the need for expanded recruitment strategies incorporating diverse demographic groups such as parent-child dyads, teacher-student pairs, and supervisor-subordinate relationships, while systematically examining potential moderating variables including gender effects. Second, the restricted optode configuration limited our measurements to the right prefrontal cortex and temporoparietal regions; future investigations would benefit from implementing bilateral fNIRS montages to achieve comprehensive whole-brain coverage. Third, the inherent spatial resolution constraints of fNIRS technology permit only macroscopic localization of cortical activity; combining fNIRS with EEG hyperscanning could significantly enhance both spatial and temporal precision. Furthermore, the absence of a control condition necessitates the implementation of active control groups to establish intervention specificity. Additionally, the lack of physiological monitoring should be addressed by integrating multimodal assessment approaches incorporating physiological measures and automated facial expression analysis to comprehensively capture emotional responses. Future studies should also pursue larger sample sizes to enable meaningful subgroup analyses and directly compare music-assisted psychodrama with conventional verbal psychodrama to elucidate their distinct neurobehavioral mechanisms.

Notwithstanding its limitations, this study provides important preliminary evidence elucidating the neural mechanisms through which music psychodrama facilitates therapeutic change. By characterizing activity patterns in specific neural systems engaged during role reversal and musical improvisation, our work advances the field from theoretical speculation to quantitative empirical support, establishing a neurobiological basis for its clinical efficacy. The neuroevidence framework developed here not only informs the precision refinement of music psychodrama interventions but also supports its targeted application—such as paired counseling sessions or scenario-based group training for students with social anxiety in educational settings. These applications leverage the strengths of musical improvisation in real-time emotional expression, integrated with dramatic elements to achieve interactivity, experiential learning, and pedagogical value. Furthermore, this study introduces an innovative experimental paradigm for the field, paving the way for deeper investigation into its neural mechanisms and the systematic dissection of core component contributions. Together, these advances lay a theoretical and practical foundation for building more efficient, neuroscience-informed psychological intervention systems.

## 5. Conclusions

This study utilized fNIRS hyperscanning to examine the neural underpinnings of role reversal within improvisational music psychodrama. The findings revealed that, relative to role-playing, role reversal elicited significantly stronger activation in the right frontal pole region and increased inter-brain synchronization in the right supramarginal gyrus. These neurodynamic patterns imply that role reversal engages both prefrontal regulatory circuits and mirror neuron systems, which collectively facilitate emotional regulation and empathic processes. Right frontal pole activation may reflect cognitive control and metacognitive monitoring during perspective-taking, while synchrony in the supramarginal gyrus suggests enhanced sensorimotor integration and mutual understanding between participants. From a clinical perspective, these results provide a neurophysiological basis for the efficacy of music psychodrama in fostering interpersonal attunement and emotional flexibility. Future research should seek to validate these preliminary observations in larger, more gender-diverse samples, employ bilateral cortical coverage to capture whole-brain dynamics, and incorporate multimodal imaging—such as combined fNIRS-EEG—to improve spatiotemporal resolution. Additionally, exploring the applicability of these mechanisms across various clinical and educational contexts would help to establish evidence-based guidelines for implementing music psychodrama in real-world settings.

## Data Availability

The original contributions presented in this study are included in the article. Further inquiries can be directed to the corresponding author.

## Appendix A

**Table A1:** The MNI coordinates and probabilistic cortical localization of all 20 channels. The corresponding Brodmann areas were identified using a brain atlas. Individual channels could cover multiple brain regions, with the sum of probabilities for overlapping areas not exceeding 1. Only channels covering more than 10% of a brain area were reported.

Channels	MNI x	MNI y	MNI z	Brodmann’s Area	*P*
CH01	12	59	40	9—Dorsolateral prefrontal cortex	0.82731
CH02	13	73	15	10—Frontopolar area	1
CH03	42	29	50	8—Includes Frontal eye fields	0.93886
CH04	20	46	50	8—Includes Frontal eye fields	0.91266
CH05	24	65	26	10—Frontopolar area	0.92015
CH06	28	69	10	10—Frontopolar area	1
CH07	52	28	39	9—Dorsolateral prefrontal cortex	0.67993
CH08	34	49	39	9—Dorsolateral prefrontal cortex	0.891
CH09	37	65	10	10—Frontopolar area	1
CH10	45	49	15	10—Frontopolar area	0.53648
CH11	64	−64	6	6—Pre-Motor and Supplementary Motor Cortex	0.97193
CH12	66	8	19	6—Pre-Motor and Supplementary Motor Cortex	0.45307
CH13	70	−35	19	40—Supramarginal gyrus part of Wernicke’s area	0.9125
CH14	65	–29	50	40—Supramarginal gyrus part of Wernicke’s area	0.45353
CH15	69	–9	31	6—Pre-Motor and Supplementary Motor Cortex	0.61489
CH16	71	–10	0	21—Middle Temporal gyrus	0.55882
CH17	69	–49	13	22—Superior Temporal gyrus	0.82026
CH18	61	–38	40	40—Supramarginal gyrus part of Wernicke’s area	0.65116
CH19	72	–22	42	42—Primary and Auditory Association Cortex	0.63125
CH20	73	–35	21	21—Middle Temporal gyrus	0.59621

## References

[1] Sang Z.Q., Huang H.M., Benko A., Wu Y. (2018). The spread and development of psychodrama in mainland China. Front. Psychol..

[2] Landis H., Skolnik S. (2024). Periphery to core: Scenes from a psychodrama. Soc. Work. Groups.

[3] Cruz A., Sales C.M., Alves P., Moita G. (2018). The core techniques of Morenian psychodrama: A systematic review of literature. Front. Psychol..

[4] Abeditehrani H., Dijk C., Neyshabouri M.D., Arntz A. (2021). Beneficial effects of role reversal in comparison to role-playing on negative cognitions about other’s judgments for social anxiety disorder. J. Behav. Ther. Exp. Psychiatry.

[5] Pellicciari A., Rossi F., Iero L., Di Pietro E., Verrotti A., Franzoni E. (2013). Drama therapy and eating disorders: A historical perspective and an overview of a Bolognese project for adolescents. J. Altern. Complement. Med..

[6] Somov P.G. (2008). A psychodrama group for substance use relapse prevention training. Arts Psychother..

[7] Wang Q., Ding F., Chen D., Zhang X., Shen K., Fan Y., Li L. (2020). Intervention effect of psychodrama on depression and anxiety: A meta-analysis based on Chinese samples. Arts Psychother..

[8] Blatner A. (1997). Psychodrama: The state of the art. Arts Psychother..

[9] Moreno J.J. (1999). Acting Your Inner Music: Music Therapy and Psychodrama.

[10] Moreno J.J. (1980). Musical psychodrama: A new direction in music therapy. J. Music Ther..

[11] Krøier J.K., Stige B., Ridder H.M. (2021). Non-verbal interactions between music therapists and persons with dementia. A qualitative phenomenological and arts-based inquiry. Music Ther. Perspect..

[12] Bergamin J.A. (2024). Habitually breaking habits: Agency, awareness, and decision-making in musical improvisation. Phenomenol. Cogn. Sci..

[13] Biasutti M., Frezza L. (2009). Dimensions of music improvisation. Creat. Res. J..

[14] Barrett K.C., Barrett F.S., Jiradejvong P., Rankin S.K., Landau A.T., Limb C.J. (2020). Classical creativity: A functional magnetic resonance imaging (fMRI) investigation of pianist and improviser Gabriela Montero. NeuroImage.

[15] Barrett K.C., Jiradejvong P., Jacobs L., Limb C.J. (2025). Children engage neural reward structures for creative musical improvisation. Sci. Rep..

[16] Diaz Abrahan V., Bossio M., Benítez M., Justel N. (2022). Musical strategies to improve children’s memory in an educational context. Psychol. Music.

[17] Müller V., Lindenberger U. (2019). Dynamic orchestration of brains and instruments during free guitar improvisation. Front. Integr. Neurosci..

[18] Zhang H., Yang J., Ni J., De Dreu C.K., Ma Y. (2023). Leader–follower behavioural coordination and neural synchronization during intergroup conflict. Nat. Hum. Behav..

[19] Cui X., Bryant D.M., Reiss A.L. (2012). NIRS-based hyperscanning reveals increased interpersonal coherence in superior frontal cortex during cooperation. Neuroimage.

[20] Liu Q., Cui H., Huang B., Huang Y., Sun H., Ru X., Zhang M., Chen W. (2023). Interbrain neural mechanism and influencing factors underlying different cooperative behaviors: A hyperscanning study. Brain Struct. Funct..

[21] Montague P.R., Berns G.S., Cohen J.D., McClure S.M., Pagnoni G., Dhamala M., Fisher R.E. (2002). Hyperscanning: Simultaneous fMRI during linked social interactions. Neuroimage.

[22] Yu X., Liu T., He L., Li Y. (2023). Micro-foundations of strategic decision-making in family business organisations: A cognitive neuroscience perspective. Long Range Plan..

[23] Hakim U., De Felice S., Pinti P., Zhang X., Noah J., Ono Y., Burgess P.W., Hamilton A., Hirsch J., Tachtsidis I. (2023). Quantification of inter-brain coupling: A review of current methods used in haemodynamic and electrophysiological hyperscanning studies. NeuroImage.

[24] Clark A. (2013). Whatever next? Predictive brains, situated agents, and the future of cognitive science. Behav. Brain Sci..

[25] Kube T., Schwarting R., Rozenkrantz L., Glombiewski J.A., Rief W. (2020). Distorted cognitive processes in major depression: A predictive processing perspective. Biol. Psychiatry.

[26] Lim M., Carollo A., Bizzego A., Chen S.A., Esposito G. (2024). Decreased activation in left prefrontal cortex during role-play: An fNIRS study of the psychodrama sociocognitive model. Arts Psychother..

[27] Fávero M., Sousa R., Budal-Oliveira L., Sousa-Gomes V. (2024). Psychodrama: Comprehensive review of the effectiveness of psychodrama in sexual abuse trauma. Eur. Psychol..

[28] Kipper D., Ritchie T. (2003). The effectiveness of psychodramatic techniques: A meta analysis. Group Dyn. Theory Res. Pract..

[29] Yaniv D. (2012). Dynamics of creativity and empathy in role reversal: Contributions from neuroscience. Rev. Gen. Psychol..

[30] Wu M., Cameirao J., Brown S. (2025). Role reversal enhances an understanding of the other, but not of the self. Arts Psychother..

[31] Cheng S., Wang J., Luo R., Hao N. (2024). Brain to brain musical interaction: A systematic review of neural synchrony in musical activities. Neurosci. Biobehav. Rev..

[32] Moreno J.J. (1991). Musical psychodrama in Naples. Arts Psychother..

[33] Moreno J.J. (1984). Musical psychodrama in Paris. Music Ther. Perspect..

[34] Feng K., Shen C.Y., Ma X.Y., Chen G.F., Zhang M.L., Xu B., Liu X.-M., Sun J.-J., Zhang X.-Q., Liu P.-Z. (2019). Effects of music therapy on major depressive disorder: A study of prefrontal hemodynamic functions using fNIRS. Psychiatry Res..

[35] Wong P.C., Chan A.H., Roy A., Margulis E.H. (2011). The bimusical brain is not two monomusical brains in one: Evidence from musical affective processing. J. Cogn. Neurosci..

[36] Abeditehrani H., Dijk C., Toghchi M.S., Arntz A. (2020). Integrating cognitive behavioral group therapy and psychodrama for social anxiety disorder: An intervention description and an uncontrolled pilot trial. Clin. Psychol. Eur..

[37] MacDonald R.A., Wilson G.B. (2014). Musical improvisation and health: A review. Psychol. Well-Being.

[38] Raglio A., Oasi O., Gianotti M., Rossi A., Goulene K., Stramba-Badiale M. (2016). Improvement of spontaneous language in stroke patients with chronic aphasia treated with music therapy: A randomized controlled trial. Int. J. Neurosci..

[39] Kipper D.A. (2002). The cognitive double: Integrating cognitive and action techniques. J. Group Psychother. Psychodrama Sociom..

[40] Ames D.L., Jenkins A.C., Banaji M.R., Mitchell J.P. (2008). Taking another person’s perspective increases self-referential neural processing. Psychol. Sci..

[41] Majdandžić J., Amashaufer S., Hummer A., Windischberger C., Lamm C. (2016). The selfless mind: How prefrontal involvement in mentalizing with similar and dissimilar others shapes empathy and prosocial behavior. Cognition.

[42] Yaniv D. (2011). Revisiting Morenian psychodramatic encounter in light of contemporary neuroscience: Relationship between empathy and creativity. Arts Psychother..

[43] Penagos-Corzo J.C., Cosio van-Hasselt M., Escobar D., Vázquez-Roque R.A., Flores G. (2022). Mirror neurons and empathy-related regions in psychopathy: Systematic review, meta-analysis, and a working model. Soc. Neurosci..

[44] Beaty R.E. (2015). The neuroscience of musical improvisation. Neurosci. Biobehav. Rev..

[45] Sasaki M., Iversen J., Callan D.E. (2019). Music improvisation is characterized by increase EEG spectral power in prefrontal and perceptual motor cortical sources and can be reliably classified from non-improvisatory performance. Front. Hum. Neurosci..

[46] Zhang Y., Meng T., Yang Y., Hu Y. (2020). Experience-dependent counselor-client brain synchronization during psychological counseling. eNeuro.

[47] Xu M., Morimoto S., Hoshino E., Suzuki K., Minagawa Y. (2023). Two-in-one system and behavior-specific brain synchrony during goal-free cooperative creation: An analytical approach combining automated behavioral classification and the event-related generalized linear model. Neurophotonics.

[48] Wang X., Wang X., Ma H. (1999). Manual of Mental Health Rating Scales.

[49] Leary M.R. (1983). Social anxiousness: The construct and its measurement. J. Pers. Assess..

[50] Zimeo Morais G.A., Balardin J.B., Sato J.R. (2018). fNIRS Optodes’ Location Decider (fOLD): A toolbox for probe arrangement guided by brain regions-of-interest. Sci. Rep..

[51] De Witte S., Klooster D., Dedoncker J., Duprat R., Remue J., Baeken C. (2018). Left prefrontal neuronavigated electrode localization in tDCS: 10–20 EEG system versus MRI-guided neuronavigation. Psychiat. Res. Neuroim..

[52] Zhang D., Zhou Y., Yuan J. (2018). Speech prosodies of different emotional categories activate different brain regions in adult cortex: An fNIRS study. Sci. Rep..

[53] Hou X., Zhang Z., Zhao C., Duan L., Gong Y., Li Z., Zhu C. (2021). NIRS-KIT: A MATLAB toolbox for both resting-state and task fNIRS data analysis. Neurophotonics.

[54] Nguyen T., Schleihauf H., Kayhan E., Matthes D., Vrtička P., Hoehl S. (2020). The effects of interaction quality on neural synchrony during mother-child problem solving. Cortex.

[55] Lu K., Yu T., Hao N. (2020). Creating while taking turns, the choice to unlocking group creative potential. NeuroImage.

[56] Cui X., Bray S., Reiss A.L. (2010). Functional near infrared spectroscopy (NIRS) signal improvement based on negative correlation between oxygenated and deoxygenated hemoglobin dynamics. Neuroimage.

[57] Emberson L.L., Crosswhite S.L., Goodwin J.R., Berger A.J., Aslin R.N. (2016). Isolating the effects of surface vasculature in infant neuroimaging using short-distance optical channels: A combination of local and global effects. Neurophotonics.

[58] Zhang M., Yin Z., Zhang X., Zhang H., Bao M., Xuan B. (2024). Neural mechanisms distinguishing two types of cooperative problem-solving approaches: An fNIRS hyperscanning study. NeuroImage.

[59] Wang H.Y., You H.L., Song C.L., Zhou L., Wang S.Y., Li X.L., Liang Z.-H., Zhang B.-W. (2024). Shared and distinct prefrontal cortex alterations of implicit emotion regulation in depression and anxiety: An fNIRS investigation. J. Affect. Disord..

[60] Grinsted A., Moore J.C., Jevrejeva S. (2004). Application of the cross wavelet transform and wavelet coherence to geophysical time series. Nonlin. Process. Geophys..

[61] Yin Z., Xuan B., Liu C., Yi J., Zheng X., Zhang M. (2025). The influence of task and interpersonal interdependence on cooperative behavior and its neural mechanisms. npj Sci. Learn..

[62] Nozawa T., Sasaki Y., Sakaki K., Yokoyama R., Kawashima R. (2016). Interpersonal frontopolar neural synchronization in group communication: An exploration toward fNIRS hyperscanning of natural interactions. Neuroimage.

[63] Fonzo G.A., Goodkind M.S., Oathes D.J., Zaiko Y.V., Harvey M., Peng K.K., Weiss M.E., Thompson A.L., Zack S.E., Mills-Finnerty C.E. (2017). Selective effects of psychotherapy on frontopolar cortical function in PTSD. Am. J. Psychiatry.

[64] Zhang Z., Olszewska-Guizzo A., Husain S.F., Bose J., Choi J., Tan W., Wang J., Tran B.X., Wang B., Jin Y. (2020). Brief relaxation practice induces significantly more prefrontal cortex activation during arithmetic tasks comparing to viewing greenery images as revealed by functional near-infrared spectroscopy (fNIRS). Int. J. Environ. Res. Public Health.

[65] Wolf R.C., Walter H., Vasic N. (2010). Increasing contextual demand modulates anterior and lateral prefrontal brain regions associated with proactive interference. Int. J. Neurosci..

[66] Yokoyama O., Miura N., Watanabe J., Takemoto A., Uchida S., Sugiura M., Horie K., Sato S., Kawashima R., Nakamura K. (2010). Right frontopolar cortex activity correlates with reliability of retrospective rating of confidence in short-term recognition memory performance. Neurosci. Res..

[67] Macuga K.L., Frey S.H. (2011). Selective responses in right inferior frontal and supramarginal gyri differentiate between observed movements of oneself vs. another. Neuropsychologia.

[68] Yin Z., Xuan B., Zhang M. (2025). How role reversal and interpersonal closeness shape verbal communication cooperation: An fNIRS hyperscanning study. Brain Struct. Funct..

[69] Allen R. (2013). Free improvisation and performance anxiety among piano students. Psychol. Music.

[70] Fachner J., Gold C., Erkkilã J. (2013). Music therapy modulates fronto-temporal activity in rest-EEG in depressed clients. Brain Topogr..

[71] Vidanagamage S.D., Bhaumik A.O., Irugalbandara A.I. (2024). Role reversal in psychodrama: Enhancing empathy and emotional understanding among institutionalized children in Sri Lanka. Int. J. Res. Innov. Soc. Sci..

[72] Usluoglu F. (2023). The effects of psychodrama on relationship between the self and others: A case study. Curr. Psychol..

[73] Toeman Z. (1946). Clinical psychodrama: Auxiliary ego double and mirror techniques. Sociometry.

[74] Belden A., Zeng T., Przysinda E., Anteraper S.A., Whitfield-Gabrieli S., Loui P. (2020). Improvising at rest: Differentiating jazz and classical music training with resting state functional connectivity. Neuroimage.

[75] Schulte-Rüther M., Markowitsch H.J., Fink G.R., Piefke M. (2007). Mirror neuron and theory of mind mechanisms involved in face-to-face interactions: A functional magnetic resonance imaging approach to empathy. J. Cogn. Neurosci..

